# Dysesthesia and Depression in Undiagnosed Alzheimer's Disease

**DOI:** 10.7759/cureus.40264

**Published:** 2023-06-11

**Authors:** Harrison J Klein, Christine M Lomiguen, Seth Carter

**Affiliations:** 1 Medical Education, Lake Erie College of Osteopathic Medicine, Erie, USA; 2 Pathology, Lake Erie College of Osteopathic Medicine, Erie, USA; 3 Family Medicine, LECOM (Lake Erie College of Osteopathic Medicine) Health Millcreek Community Hospital, Erie, USA; 4 Geriatrics, LECOM (Lake Erie College of Osteopathic Medicine) Health Institute for Successful Aging, Erie, USA; 5 Geriatrics, Lake Erie College of Osteopathic Medicine, Erie, USA; 6 Geriatrics, LECOM (Lake Erie College of Osteopathic Medicine) Health Millcreek Community Hospital, Erie, USA

**Keywords:** alzheimer's, depression, geriatrics, dementia, alzheimer disease, dyesthesia

## Abstract

Dysesthesia is an abnormal sensation typically described as tingling, burning, or itching. Dysesthesia may occur in the presence or absence of causative dermatologic or medical pathology. Sensory abnormalities have been well documented in patients suffering from Alzheimer's disease (AD), though reported abnormalities typically affect the olfactory, visual, and auditory systems. Research describing dysesthetic symptoms in AD is scarce. Major depressive disorder (MDD) is another phenomenon commonly associated with AD, though depressive symptoms are frequently masked by cognitive deficits. Less attention has been given to the reverse of this relationship, in which MDD masks the symptoms of AD. Here, we present a case of undiagnosed AD in a geriatric patient presenting with primary complaints of depression and dysesthesia. We then discuss the pathophysiology of dysesthesia, MDD, and AD, as well as how these entities may interact in a single patient. Lastly, we emphasize the importance of thorough history-taking and multi-specialty collaboration in the care of older adults.

## Introduction

Dysesthesia is an abnormal sensation typically described as tingling, burning, or itching [1-2]. This phenomenon is often secondary to discrete lesions within the nervous system caused by trauma, irritation, or impingement. Dysesthesia can also be caused by more systemic etiologies, including metabolic derangements (diabetes mellitus, hypothyroidism, nutritional deficiency), infections (human immunodeficiency virus, varicella zoster virus), toxins (chemotherapeutics, statins, antimicrobials, immunosuppressants), and hereditary disorders (metachromatic leukodystrophy, Charcot-Marie-Tooth disease) [1-3]. 

Cutaneous sensory disorder (CSD) is characterized by dysesthetic sensations in the absence of causative dermatologic or medical pathology. Coexisting neurologic and psychiatric disorders are common, including stroke, major depressive disorder (MDD), post-traumatic stress disorder (PTSD), and anxiety disorders [4]. Research has suggested that sensory impairments may also be associated with Alzheimer's disease (AD), but current literature does not specifically associate CSD symptoms with AD [5].

MDD is a psychiatric disorder that commonly presents to the primary care physician under the guise of varied complaints. These include a lack of energy, generalized pain, headaches, or, in some instances, dysesthesia. Special attention should be given to high-risk groups for the development of MDD, including older adults and those who suffer from chronic disease [4,6]. Previous studies have supported a low index of suspicion for MDD in the elderly, as depressive symptoms are often ascribed to cognitive impairment [7]. Further complicating diagnosis is the bi-directionality of this relationship, in which the cognitive impairments of dementia are mistaken for depressive symptoms. In fact, following apathy, depressed mood is the second most common neuropsychiatric symptom of AD [8]. Late-life MDD and dementia are thus exceptionally difficult to distinguish.

Here, we present the case of a 74-year-old male who presented to the clinic with a recurrent abnormal sensation of internal body heat and treatment-resistant depression. The patient consulted endocrinology, neurology, psychiatry, and geriatrics. While still living, it was concluded that his symptoms were psychosomatic in nature and likely a result of his MDD. Upon autopsy, however, the patient was diagnosed with AD. This case highlights the importance of thorough history-taking and encourages clinicians to have heightened suspicion for AD in geriatric patients with depression and dysesthesia.

## Case presentation

This patient was a 74-year-old male with a medical history significant for type 2 diabetes mellitus, hypertension, MDD, and anxiety who presented to the endocrinologist upon referral from his primary care physician for consultation and management of an abnormal sensation of internal body heat. The patient’s wife, who was present for all of his appointments, reported that this sensation first occurred after receiving electroconvulsive therapy (ECT) for treatment-resistant depression. His most recent ECT session was one year prior to visiting the endocrinologist. The warmth sensation typically occurred when supine at night and resolved once he sat upright, ambulated, or drank cold water. This caused significant sleep disruption. The episodes of warmth were resistant to both ibuprofen and acetaminophen. The patient reported that his temperature was usually 97º F during episodes and denied feeling warm to the touch, though admitted to chest flushing. He endorsed a long-standing loss of appetite, with an involuntary hospitalization occurring one month prior to his endocrinology appointment due to not eating for 48 hours. There was an associated 12-pound weight loss over the six weeks before his evaluation. He admitted to fatigue and “drinking water all day” but denied polyuria, hair loss, chest pain, palpitations, abdominal pain, nausea, vomiting, diarrhea, and constipation. He was a never-smoker and did not consume alcohol or caffeine. The patient was evaluated by a neurologist prior to presentation with no noted abnormalities. Surgical history was significant for cholecystectomy and hydrocelectomy. His medications included metformin 500 mg by mouth daily, lisinopril 20 mg by mouth twice daily, tamsulosin 0.8 mg by mouth daily, and amlodipine 10 mg by mouth daily. Treatment with olanzapine 5 mg and sertraline 50 mg by mouth daily was initiated one year prior to the endocrinology appointment, though discontinued after several months for unknown reasons. He restarted this combination two weeks prior to his appointment without symptom relief. 

The endocrinologist determined there was no obvious etiology for the patient’s symptoms and that ECT was unlikely to be associated with his symptoms. Additional labs were obtained, including a complete blood count (CBC), comprehensive metabolic panel (CMP), thyroid stimulating hormone (TSH), thyroxine (free T4), anti-thyroid peroxidase, anti-thyroglobulin antibodies, thyroid stimulating immunoglobulin (TSI), cortisol (A.M.), and an aldosterone/plasma renin activity ratio. In the meantime, gabapentin 400 mg was added to his medication regimen by the endocrinologist. TSH and free T4 were both found to be normal. There was an elevated anti-thyroglobulin antibody titer but a normal anti-thyroid peroxidase antibody titer. His aldosterone/renin ratio was elevated, prompting an order for bilateral renal ultrasonography. The sonogram demonstrated bilateral renal cortical atrophy with a simple exophytic cyst in the right kidney, but otherwise negative for pathology. CBC, CMP, and cortisol (A.M.) were within normal limits. 

The patient was then referred to geriatrics by his endocrinologist for a second opinion on the management of his depression and dysesthesia. The olanzapine 5 mg and sertraline 50 mg were continued and pharmacogenetic testing was ordered. At the two-week follow-up, no improvement in depressive symptoms was noted, prompting an increase in sertraline strength to 100 mg. The patient continued to endorse fatigue and weakness, now with the sensation of lightheadedness. He also admitted to a weak stream while urinating, but denied dysuria. He reported drinking larger volumes of water to help with the dysesthesia, which eventually led to frequent urination. An electrocardiogram, cardiac stress test, and prostate-specific antigen (PSA) level were all within normal limits. He returned to the geriatrician two weeks later and discontinued olanzapine 5 mg due to a lack of improvement in depressive symptoms and concern for side effects. 

On follow-up three weeks later, the patient reported that the warmth episodes began to occur in the morning and afternoon along with the typical nighttime pattern. They were also accompanied by pain during this time period and exacerbated by loud noises. His psychiatrist recommended weaning sertraline 100 mg and gabapentin 400 mg due to ineffectiveness. Several weeks later, he returned to the geriatrician for further evaluation at the request of his wife. However, after becoming increasingly frustrated with the uncertainty, he declined to pursue further testing with regard to his abnormal sensations of warmth. At this visit, it was noted that the patient relied more and more upon the use of an independent historian to recall medical information and had undergone substantial weight loss secondary to a loss of appetite. These were thought to have been related to increased depression and anxiety caused by the worsening dysesthesia.

**Figure 1:**
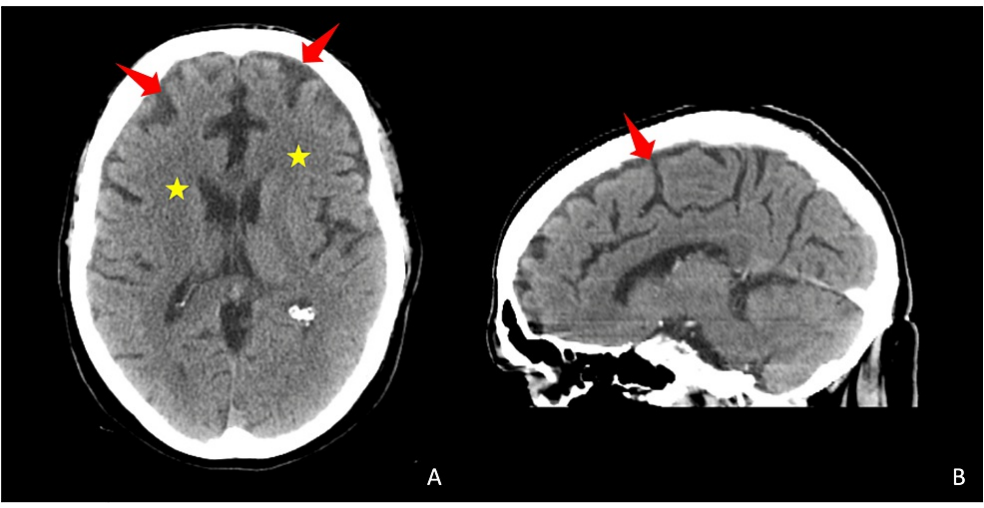
Transverse (A) and sagittal (B) slices obtained from computed tomography (CT) of the head with intravenous contrast. Global brain atrophy (arrow) and chronic ischemic changes (star) are notable in this scan. There is no evidence of an acute intracranial process.

Several weeks later, the patient’s wife convinced him to undergo a CT scan of the head with intravenous contrast (Figure 1). This was significant for global brain atrophy and chronic ischemic changes. Several weeks following his CT, he was admitted to the hospital due to continued weakness with lightheadedness, fatigue, and anorexia. He passed away three days later due to acute bronchopneumonia. After his death, a histological exam of brain tissue confirmed a diagnosis of AD. 

## Discussion

Dysesthesia is currently understood as a form of neuropathic pain [9,10]. The etiology of neuropathic pain is multifactorial and includes aberrant nociceptive activity, reduction in central and peripheral stimulus threshold, impaired neuronal modulation, and inappropriate activation of microglia [10]. Tricyclic antidepressants, serotonin-norepinephrine re-uptake inhibitors, and voltage-gated calcium channel ligands are considered mainstays of treatment for neuropathic pain, such as dysesthesia [11,12]. 

Numerous differential diagnoses were entertained for this patient’s dysesthetic symptoms. The first of these was hyperthyroidism, as elevated levels of thyroxine can induce heat intolerance via adrenergic excess and hyper-metabolic activity [13]. However, his TSH and free T4 were both within normal limits. The elevated anti-thyroglobulin antibody titer and normal anti-thyroid peroxidase antibody titer were not pathognomonic for any particular thyroid disease. Another diagnosis we entertained was an adverse effect of amlodipine. Flushing occurs in approximately 2.6% of patients, likely due to the medication’s vasodilatory properties [14]. This diagnosis was less likely considering the pain and noise sensitivity reported by the patient during his episodes. Lastly, ECT was considered as a potential etiology. ECT has been documented to cause a temporary increase in blood pressure during the procedure and in the immediate recovery period [15]. This patient's pattern of relapsing and remitting sensations was not compatible with this reported effect of ECT. 

Our patient demonstrated a unique combination of dysesthesia and occult AD with significant depressive mood symptoms. While seemingly unrelated, a review of contemporary neurological research has revealed AD as a potential explanation for his depressive and sensory symptoms. Sensory alterations have been well documented in patients with AD, particularly disturbances in olfaction [8]. For example, Roberts and colleagues identified an association between olfactory impairment and amnestic mild cognitive decline (aMCI) [16]. Olfactory impairment was even demonstrated to predict progression from aMCI to AD [16]. Auditory and visual impairments have also received attention as possible AD screening modalities; however, evidence for these methods is less robust [8]. Unfortunately, the vast majority of AD literature focuses on olfactory, visual, and auditory impairment rather than dysesthetic symptoms. This highlights an important research gap that requires careful investigation. 

This patient's depressive symptoms may have also been mediated by AD. Depressed mood is the second most common neuropsychiatric symptom experienced by AD patients and may be a hallmark of prodromal AD [7]. Despite this high prevalence, MDD can be under-diagnosed in geriatric patients with dementia. This is due to the frequently atypical symptoms of late-life depression and the masking of depressive symptoms by cognitive deficits [17]. An interesting facet of this case is the reversal of roles between AD and depression. As described previously, dementia often masks depression and may delay diagnosis in affected patients. However, this patient's long-standing MDD diagnosis acted as an anchor to numerous physicians, preventing the proper recognition of his depression as a neuropsychiatric manifestation of early AD. He was also very skilled at masking the other symptoms of AD by taking comprehensive notes, calendaring, and maintaining a rigid routine during the early years of the disease. An important limitation of this case study is that this patient's AD diagnosis relies entirely on post-mortem examination. No formal neurocognitive testing was administered while the patient was still alive. 

## Conclusions

Depression and sensory abnormalities are associated with AD in the geriatric population. While dementia typically masks the symptoms of depression, it is important to recognize the reverse is also true. Thus, elderly patients presenting with treatment-resistant depression should be carefully evaluated for signs of dementia. Current evidence most strongly supports the use of auditory, visual, and olfactory impairments as indicators of prodromal or fulminant dementia. It may be worth investigating the frequency of dysesthesia presenting in AD patients. This case highlights the importance of thorough history taking and low threshold of suspicion for AD in geriatric patients presenting with treatment-resistant depression and dysesthesia.
